# Current landscape and comprehensive management of glycemic variability in diabetic retinopathy

**DOI:** 10.1186/s12967-024-05516-w

**Published:** 2024-07-29

**Authors:** Bo Chen, Chaozan Shen, Bao Sun

**Affiliations:** 1 Department of Pharmacy, The Central Hospital of Yongzhou, Yongzhou, China; 2 Department of Clinical Pharmacy, The Second People’s Hospital of Huaihua, Lulin Road, Huaihua, Hunan 418000 China; 3 Department of Pharmacy, The Second Xiangya Hospital, Central South University, No.139 Middle Renmin Road, Changsha, Hunan 410011 China; 4 Institute of Clinical Pharmacy, Central South University, Changsha, China

**Keywords:** Diabetic retinopathy, Glycemic variability, Diabetes-related complications, Relevant mechanisms, Mechanism-based therapeutic strategies

## Abstract

Diabetic retinopathy (DR), a well-known microvascular complication of diabetes mellitus, remains the main cause of vision loss in working-age adults worldwide. Up to now, there is a shortage of information in the study regarding the contributing factors of DR in diabetes. Accumulating evidence has identified glycemic variability (GV), referred to fluctuations of blood glucose levels, as a risk factor for diabetes-related complications. Recent reports demonstrate that GV plays an important role in accounting for the susceptibility to DR development. However, its exact role in the pathogenesis of DR is still not fully understood. In this review, we highlight the current landscape and relevant mechanisms of GV in DR, as well as address the mechanism-based therapeutic strategies, aiming at better improving the quality of DR management in clinical practice.

## Introduction

Diabetic retinopathy (DR), a well-known microvascular complication of diabetes mellitus, is the leading cause of preventable blindness among adults aged 20–74 years [1–3]. Classified as non-proliferative DR (NPDR) and proliferative DR (PDR), both of which can cause diabetic macular edema, DR is the most common and serious ocular complication. Currently, the global prevalence of DR is still high, affecting 2 in every 10 patients with diabetes, particularly in low-to-middle-income countries, and the global DR burden is expected to remain high through 2045 [4]. Apart from its worsening effects on vision, DR also signifies a increasing risk of life-threatening cardiovascular complications [5].

Although multi-level risk factors including poor glycemic control, disease duration, hypertension, education levels, low-density lipoprotein cholesterol, multiple dietary intakes and low socioeconomic status were considered to be associated with the presence and the severity of DR across diverse geographies [6–10], there was a lack of consensus viewpoint regarding the effect of glycemic levels mainly reflected by hemoglobin A1c (HbA1c) on DR. For instance, a previous study showed that patients treated with ranibizumab acquired improvement in DR severity score seemingly to be independent of baseline HbA1c [11]. Therefore, apart from HbA1c, other factors might be related to development of DR.

In recent years, increasing evidence addressed that glycemic variability (GV), referred to the degree of blood glucose fluctuation, could be an additional risk factor for the progress of DR [12–15]. A prospective cohort study including 2,005 patients with T2DM revealed that high visit-to-visit fasting plasma glucose (FPG) variability was related to new-onset PDR and diabetic macular oedema [12]. Hsing et al. assessed the effect of glycemic gaps on DR progression and found that GV had deleterious effects on DR progression [13]. Similarly, the Diabetes Control and Complications Trial (DCCT) study compared the effect of the “intensive” treatment group (mean HbA1c of 7.2%) and the “conventional” group (mean HbA1c of 9.2%) on DR in 1,441 patients with type 1 diabetes mellitus (T1DM) and concluded that earlier tight control could lower the updated mean HbA1c and seemed to be more beneficial for reducing the risk of DR [16]. Inconsistently, a recent real-world study considered that the rapid reduction of HbA1c was not involved in the progression of mild or moderate NPDR [17]. Thus, the role of GV in the development of DR remains elusive, lacking a unified definition and consensus. The aim of this study is to highlight the state-of-the-art knowledge regarding the role and relevant mechanisms of GV in DR, as well as address the mechanism-based strategies, aiming at better improving the quality of DR management in clinical practice.

## Measurements and variables for GV

Since HbA1c cannot reflect the quantification of glycemic value fluctuations, GV defined by the measurement of fluctuations within a given time interval attracts increasing interest in evaluating the overall quality of glycemic oscillations. However, there is little consensus on the standard method to assess GV [18]. The traditional method to measuring GV relied on self-monitoring of blood glucose (SMBG) [19], which measured seven daily glucose values via glucose meters and lacked essential information regarding glycemic oscillations between the measurements. Continuous glucose monitoring (CGM), including real-time CGM (rtCGM) and intermittently viewed CGM (iCGM), addressed many of the limitations inherent in self-monitoring of blood glucose [20]. Characterized by the utilization of subcutaneous sensors to measure glucose with interstitial glucose measurements at 5 min intervals, CGM provided feedback to the individual about glucose levels and trends [21–24]. Apart from the CGM, flash glucose monitoring (FGM) was also reported to be a new approach to glucose monitoring, which indicated direction and speed of glucose change with a long sensor lifetime of 14 days and emerged as a practical solution to the glucose monitoring [25, 26]. In short, measurements used for evaluating GV represent a critical issue and have certain characteristics, which should be taken into account when effectively evaluating the metrics that quantify the GV.

**Fig. 1:**
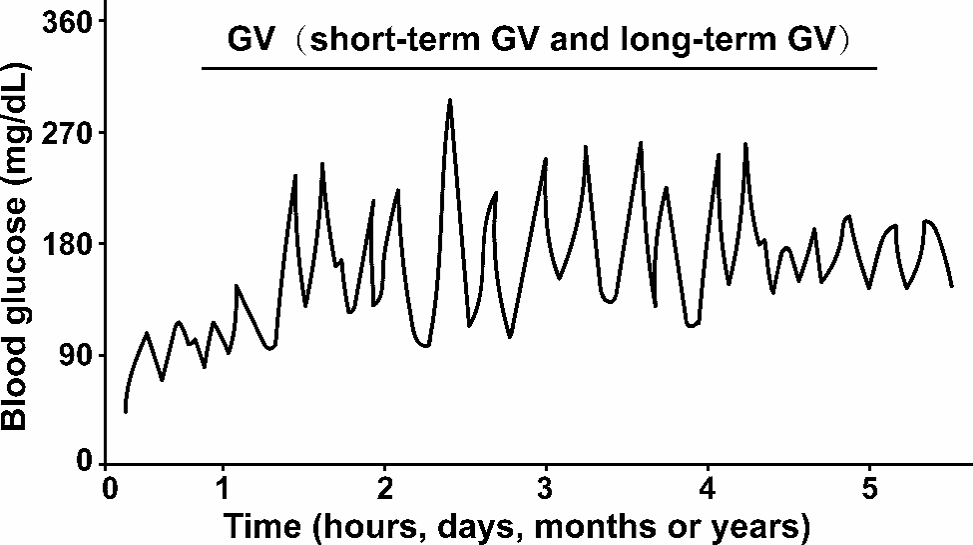
GV mainly includes two categories of metrics based on the length of time-interval: long-term GV and short-term GV. Long-term GV is commonly based on visit-to-visit glycemic excursions of FPG, PPG and HbA1c over a month or year. Short-term GV is characterized by sudden and rapid glucose changes, primarily reflecting glycemic excursions within hours or days

Presently, although a definitive consensus on the optimal approach for measuring GV in clinical practice has yet to be established, various variables or metrics quantifying GV have been proposed with the continuous advancement in the accuracy and complexity of CGM devices [27, 28] (Table 1). Until recently, there are predominantly two categories of metrics based on the length of time-interval: long-term GV (months to years) and short-term GV (hours to days) (Fig. 1). Long-term GV is commonly based on visit-to-visit glycemic excursions of FPG, postprandial glucose (PPG) and HbA1c over a month or year [29], with the subsequent calculation of their standard deviation (SD) and coefficient of variation (CV), and reflects the variation around the mean value of blood glucose, FPG, PPG and HbA1c between sequential visits [19, 30]. In addition to CV and SD, successive variation (SV), variation independent of the mean (VIM), and average real variability (ARV) are also the metrics of long-term GV [31, 32]. The SV is calculated as the square root of the mean square variance between sequential measurements [33]; the VIM is calculated as the SD divided by the mean to the power x, with x derived from a fitting nonlinear regression model [34], and the ARV is calculated as the average of the absolute differences between successive glycemia measurements [35].

**Table 1:** Various variables or metrics quantifying GV

Variables or metrics of GV	Definition or computation	References
Long-term GV		
SD and CV	Variation of blood glucose values (FPG, PPG and HbA1c) between sequential visits (months to years)	[19, 30]
SV	Calculated as the square root of the mean square variance between sequential measurements	[33]
VIM	Calculated as the SD divided by the mean to the power x, with x derived from a fitting nonlinear regression model	[34]
ARV	Calculated as the average of the absolute differences between successive glycemia measurements	[35]
Short-term GV		
SD and CV	Intraday/interday variation of blood glucose values (FPG, PPG and HbA1c)	[27]
IQR	The distribution of glucose data at given timepoints	[36–38]
MAGE	Calculated as the mean distances between consecutive peaks and nadirs of blood glucose	[40]
CONGA	Obtained as the SD of differences between the pre-defined time intervals through spectral analysis	[42]
TIR	Defined as the percentage of time that individuals could maintain their glucose exposure within the target glucose range of 3.9–10.0 mmol/L	[43]
MODD	Calculated as the mean absolute differences between glycemia at the same time within a 24 h interval	[44]
HBGI/LBGI	Measuring the area under the curve when the blood glucose value was above/below a predetermined value	[45, 46]
ADRR	Representing the daily sum of peak risks for hypo- and hyperglycemia	[47]

GV: glycemic variability; SD: standard deviation; CV: coefficient of variation; FPG: fasting plasma glucose; PPG: postprandial glucose; HbA1c: hemoglobin A1c; SV: successive variation; VIM: variation independent of the mean; ARV: average real variability; IQRs: interquartile ranges; MAGE: mean amplitude of glycemic excursions; CONGA: continuous overlapping net glycemic action; TIR: time in range; MODD: mean of daily differences; HBGI: high blood glucose index; LBGI: low blood glucose index; ADRR: average daily risk range

Short-term GV as another established metric of GV is characterized by sudden and rapid glucose changes, mainly including within-day and between-day GV. SD and CV are the common metrics of not only long-term GV, but also short-term GV. In recent years, interquartile range (IQR), an important indicator in the ambulatory glucose profile, was proposed as a powerful tool for assessing GV, primarily reflecting interday variability [27, 36–38]. Besides, there existed robust correlations between average IQR and within-day GV metrics, such as SD and CV [39]. The mean amplitude of glycemic excursions (MAGE) was calculated as the mean distances between consecutive peaks and nadirs of blood glucose [40], which mainly provided insights regarding the extent to which glycemic excursions occur, particularly accounting for fasting state hypoglycemia and postprandial hyperglycemia [41]. As a novel measurement of GV, the Continuous Overall Net Glycemic Action Index (CONGA) was obtained as the SD of differences between the pre-defined time intervals through spectral analysis [42], which has flexibility in the time interval for analyzing GV, thus fitting better in the individual clinical scenario. More recently, time in range (TIR) was defined as the percentage of time in which individuals could maintain their glucose exposure within the target glucose range of 3.9–10.0 mmol/L [43], which was currently method to evaluate glycemic control in the standard of care and provided the information on short-term GV. The mean of daily differences (MODD) was calculated as the mean absolute differences between glycemia at the same time within a 24 h interval, which was considered to be the standard index for estimating the between-day GV [44]. To quantify the risk of hyperglycemic episodes, the high blood glucose index (HBGI) was introduced via measuring the area under the curve when the blood glucose value was above a predetermined value [45]. On the contrary, the low blood glucose index (LBGI) was identified through measuring the area under the curve when the blood glucose value was below a predetermined range [46]. Average daily risk rates (ADRR) represented the daily sum of peak risks for hypo- and hyperglycemia [47], which could serve as a reliable predictor of excessive glucose levels. Altogether, the variables used for evaluating GV remain a critical issue and have certain limitations, which should be taken into account when interpreting studies investigating the association of GV with DR.

Despite various indicators that evaluated the glycemic fluctuations in different time intervals, there was little consensus on the standard target for GV. The Advanced Technologies & Treatments for Diabetes International Consensus recommended a CV threshold of 36% in clinical practice [48], and studies found that individuals with a CV > 36% experienced three- to six-fold higher risk of hypoglycemic events compared with those with a CV of ≤ 36% [22, 48]. Notably, the Chinese researchers identified a CV threshold of 33% in patients with T2DM, which could define excessive variability [49, 50]. On the other hand, numerous studies demonstrated that GV metrics, including CV, SMBG, TIR and mean glucose, were solidly associated with the frequency of scanning by continuous glucose monitoring sensors [51–53], while they have not been linked to microvascular complications in prospective studies [54]. Furthermore, no robust association of CV with other glucose metrics, such as HBGI and TIR, was observed in real-world studies with large patient cohorts [55, 56], although it appeared to have utility in predicting hypoglycemia [57, 58]. These findings emphasize that further studies are necessary to clarify the optimal frequency of scanning by continuous glucose monitoring sensors and the optimal target for GV in clinic.

## Landscapes and relevant mechanisms of GV in DR

Emerging studies and a series of meta-analysis have shown that GV plays an important role in diabetic complications, such as DR [29, 59–61] (Table 2), and may be useful to predict DR progression in clinical practice. An earlier study explored the association of HbA1c variability with risk of microvascular complications in 1,706 patients with T1DM and found that SD of HbA1c was correlated with early DR [62]. Similarly, another study including 220 patients with T1DM revealed that both mean HbA1c and HbA1c-CV were independently associated with DR [63]. Consistent with this result, Mao et al. also showed a significant independent association of HbA1c variability with the risk of microvascular complications, including DR in T1DM patients [64]. Using the DCCT data set, Beck et al. demonstrated that for each 10% points lower in TIR, the hazard rate of development of DR progression was increased by 64% in T1DM patients [65].

**Table 2:** Roles of GV in DR

Metrics of GV	Subjects	Effects	References
SD of HbA1c	1,706 patients with T1DM	Increased the risk of early DR	[62]
Mean HbA1c and CV of HbA1c	220 patients with T1DM	Independently associated with the increased risk of DR	[63]
IQRs of HbA1c	1,240 patients with T1DM	An independently risk factor for microvascular complications, including DR	[64]
TIR	1,440 participants with T1DM	Reduced the hazard rate of development of DR progression	[65]
SD and CV of HbA1c and FPG	654 individuals with T2DM	Predicted DR progression in patients with good glycemic control	[66]
Mean HbA1c and SD of HbA1c	1,125 participants with T2DM	Associated with a higher risk of ≥ 3 step progression, and progression to PDR	[67]
CV and ARV of FPG	10,251 and 1,791 individuals with T2DM	Increased the risk for DR	[68]
CV of FPG	457 participants with T2DM	Accurately predicted the development of DR	[69]
TIR	3,262 patients with T2DM	Inversely correlated with the severity of DR	[70]
TIR	2,030 adult patients with T2DM	Increased the risk for DR	[71]
CV of FPG	6,770 individuals with T2DM	Associated with increased risk of DR	[72]
SD and CV of FPG	2,005 patients with T2DM	Correlated with the development of PDR	[12]
SD of blood glucose	2,927 patients with T2DM	An independent risk factor of DR	[73]

GV: glycemic variability; DR: diabetic retinopathy; SD: standard deviation; HbA1c: hemoglobin A1c; T1DM: type 1 diabetes mellitus; IQRs: interquartile ranges; TIR: time in range; CV: coefficient of variation; FPG: fasting plasma glucose; T2DM: type 2 diabetes mellitus; PDR: proliferative DR; FPG: fasting plasma glucose

In addition to the DR in T1DM patients, GV also played a critical role in patients with DR in T2DM. A prospective study enrolling 654 T2DM patients showed that GV estimated by SD and CV of HbA1c and FPG predicted DR progression in patients with good glycemic control (HbA1c ≤ 7.5%, 58 mmol/mol) [66]. Park et al. performed a retrospective study in 1,125 participants with T2DM and found that higher glycemic variability, including mean HbA1c and HbA1c-SD, had a higher risk of ≥ 3 step progression, and progression to PDR [67]. In both Action to Control Cardiovascular Risk in Diabetes (ACCORD) and the Veteran Affairs Diabetes Trial (VADT), the CV and ARV of FPG was related to increased risk for microvascular complications, including nephropathy and DR, even after adjusting for other risk factors [68]. Also, the Treatment Options for Type 2 Diabetes in Adolescents and Youth (TODAY) study provided the evidence that FPG variability estimated by CV during year 1 could accurately predict the development of comorbidities, such as nephropathy, and retinopathy progression over the subsequent 10 years [69]. Recently, TIR as an intuitive metric of GV was also reported to be associated with the prevalence of DR in T2DM. Lu et al. recruited 3,262 patients with T2DM and reported that TIR was significantly associated with all stages of DR, and the severity of DR was inversely correlated with TIR quartiles [70]. Another cross-sectional study including 2,030 patients showed that for each absolute 10% decrease in TIR, the risk of DR was increased by 8% [71].

Although GV was undoubtedly associated with DR, the strength of associations varied. The Hoorn Diabetes Care System cohort study enrolling 6,770 individuals with T2DM supported that a higher FPG-CV was associated with increased risk of DR; however, for HbA1c, the correlation was weaker and less consistent [72]. Likewise, the visit-to-visit FPG variability reflected by SD and CV was also found to be correlated with the development of PDR in 2,005 patients with T2DM, while neither the SD nor CV of HbA1c was associated with the development of PDR or DR [12]. On the other hand, Lu et al. observed that no metrics of GV were correlated with DR in latent autoimmune diabetes of the adult (LADA), but the SD of blood glucose values was significantly associated with DR in T2DM patients after adjusting for confounders [73]. In contrast, another observational cohort study showed that HbA1c-SD was not associated with an increased risk of DR after adjustment for sex, age, diabetes type and the mean [74]. More importantly, a prospective cohort study consisting of 315 patients with T2DM demonstrated that visit-to-visit HbA1c variability was also not significantly associated with the risk of DR [75]. Recently, a real-world study including 1,150 T2DM patients with early worsening of DR (EWDR) and 1,150 matched controls analyzed the correlation between magnitude of the reduction of HbA1c and EWDR [17]. The results provided the evidence that the rapid reduction of HbA1c was not associated with progression of mild or moderate NPDR. The plausible reasons for these inconsistent results were as follows: (1) the sample size and follow-up time of these studies was different, which limited the consistency of results; (2) the residual or potential confounders might have a critical impact on the risk of DR; (3) the included subjects lacked relevant data regarding the time of onset and staging of DR. Therefore, further large-scale longitudinal studies are necessary to identify the optimal variability measurement and investigate the definite association between GV and DR.

**Fig. 2:**
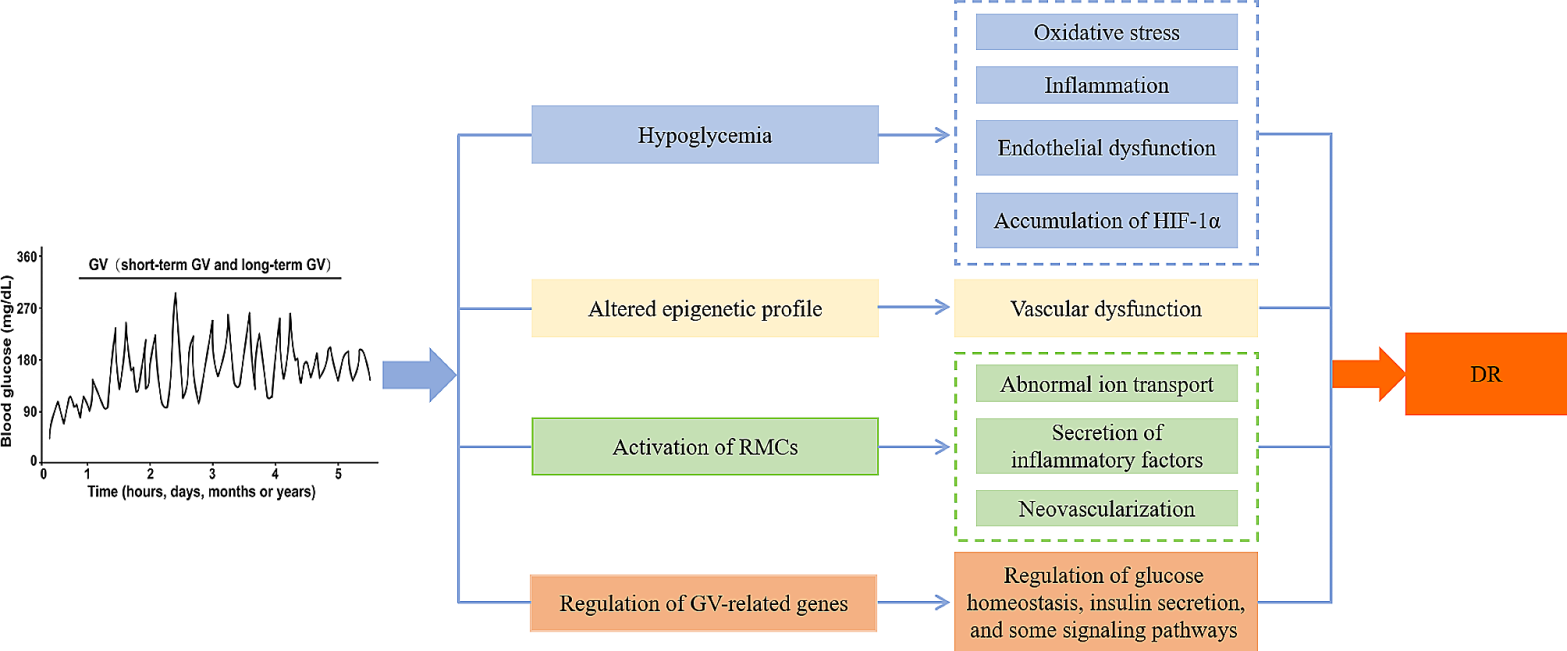
The relevant mechanisms of GV involved in DR. GV is involved in DR through hypoglycemia, epigenetics changes, activation of RMCs and regulation of GV-related genes. Among them, hypoglycemia can induce oxidative stress, inflammation, endothelial dysfunction and accumulation of HIF-1α, epigenetics changes can lead to vascular dysfunction, activation of RMCs can induce abnormal ion transport, secretion of inflammatory factors and neovascularization, and GV-related genes can regulate glucose homeostasis, insulin secretion and several signaling pathways, which ultimately contributes to DR

The relevant mechanisms of GV involved in DR were complicated. Previous studies highlighted that high GV was significantly associated with the increased risk of hypoglycemia episodes [76–78]. Numbers of studies had shown that GV and hypoglycemia might play a vital role in aggravating diabetic complications, including DR, through oxidative stress, inflammation and endothelial dysfunction [79–83]. Notably, Piconi et al. showed that both stabilization or oscillation of hyperglycemia could enhance oxidative stress generation and increase endothelial cell apoptosis via elevating the production of ROS at the mitochondrial transport chain level [84]. In addition, Costantino et al. revealed that MAGE and postprandial incremental area under the curve was independently correlated with the altered epigenetic profile on the adaptor protein p66^Shc^ (a key driver of mitochondrial oxidative stress) promoter, which might explain the vascular dysfunction in T2DM patients [85]. Activated retinal Müller cells (RMCs) identified as the main glial cells in the retina were essential for maintaining retinal homeostasis, which played an important role in the pathogenesis of DR [86]. The activation of RMCs occurred in response to glucose excursions [87], which might ultimately contribute to various pathological changes in DR, including abnormal ion transport, secretion of inflammatory factors and neovascularization [86]. In recent years, studies identified that hypoxia-inducible factor-1α (HIF-1α) as an important transcriptional regulatory factor played a crucial role in the occurrence and development of PDR [88–90]. Guo et al. showed that hypoglycemia could enhance the accumulation of HIF-1α, which resulted in the increased expression of HIF-dependent angiogenic mediators, thus promoting DR [90]. On the other hand, Saik et al. constructed the GV-related network via the bioinformatics analysis and identified several GV-related genes that were involved in the regulation of glucose homeostasis, insulin secretion, as well as some signaling pathways, which played indispensable parts in the networks of diabetes complications, including DR and cardiovascular disease [91]. Taken together, these results suggest that GV is involved in DR by inducing oxidative stress, inflammation, endothelial dysfunction and epigenetics changes, as well as regulating the activation of RMCs and relevant GV-related genes (Fig. 2).

## Therapeutic strategies for improving GV

In light of the above findings, a growing body of research underlines the therapeutic strategies for improving GV. Non-pharmacological and pharmacological therapeutic strategies are introduced for improving GV in clinical practice (Table 3).

**Table 3:** Therapeutic strategies for improving GV

Therapeutic strategies	Subjects	Results	References
Non-pharmacological strategies			
Intermittently-scanned CGM	68 T1DM patients	Significantly improved GV	[95]
FreeStyle Libre Flash CGM	Insulin-treated older adults with T2DM	Stabilized TIR and reduced TBR	[96]
Low-carbohydrate diet	27 T2DM patients	Lowered postbreakfast glucose excursions	[97]
Low-carbohydrate diet	50 T2DM patients	Increased TIR	[98]
Combined aerobic and resistance exercise training	50 sedentary diabetes patients	Improved blood glucose fluctuation	[101]
Washed microbiota transplantation	14 patients with unstable diabetes	Ameliorate GV indices	[102]
Multidisciplinary team approaches	3,060 uncontrolled T2DM patients	Remarkably associated with improvements in glycemic control	[103]
Pharmacological strategies			
Basal insulin	393 patients with T2DM	Reduced GV and hypoglycemia	[107]
Insulin degludec and glargine	60 children with T1DM	Contributed to better HbA1c and TIR with reduced hypoglycemia	[108]
Insulin degludec	36 individuals with T1DM	Contributed to a smaller day-to-day variability of FPG	[109]
DPP4i	1816 patients with T2DM	Reduced GV and ultimately contributed to the delay of insulin initiation	[110]
Vildagliptin	20 Japanese outpatients with T2DM	Contributed to a significant decrease in the median HbA1c	[111]
Anagliptin	89 patients with T2DM	Remarkably improved MAGE and TIR	[112]
Sitagliptin	340 Japanese patients with early-stage T2DM	Significantly superior in achieving TIR	[113]
Dapagliflozin as an adjuvant therapy to basal insulin	36 Japanese patients with T2DM	Significantly decreased the fluctuations of blood glucose	[114]

GV: glycemic variability; CGM: continuous glucose monitoring; T1DM: type 1 diabetes mellitus; T2DM: type 2 diabetes mellitus; TIR: time in range; TBR: time below range; HbA1c: hemoglobin A1c; DPP4i: dipeptidyl peptidase-4 inhibitors; MAGE: mean amplitude of glycemic excursions

### Non-pharmacological therapeutic strategies

Emerging systematic reviews reported that CGM could increase TIR and improve GV in adults with T1DM and T2DM [92–94]. Also, an open-label randomized control cross over study revealed that rt-CGM and intermittently-scanned CGM improved short- and long-term blood glucose control in T1DM individuals [95]. Furthermore, another prospective observational cohort study suggested that CGM could reduce GV and increase the stability of TIR in insulin-treated older adults with T2DM [96]. Interestingly, dietary carbohydrate intake has a great impact on postprandial hyperglycemia and GV. A clinical trial proved that a breakfast low in carbohydrate could significantly reduce glucose excursions and improve GV in T2DM patients [97]. Likewise, Deshmane et al. performed an interventional clinical trial and also found that.

lowering carbohydrates increased TIR in individuals with T2DM [98]. Besides, it was worth noting that physical exercises as non-pharmacological interventions were essential for improving glycemic control [99]. High-intensity interval training was proposed to ameliorate fasting blood glycemia in patients with T2DM [100]. Moreover, combined aerobic and resistance exercise training could decrease SD and CV of blood glucose and improve blood glucose fluctuation in diabetes patients [101]. Currently, a new method regarding fecal microbiota transplantation or washed microbiota transplantation was employed to ameliorate GV indices [102], raising a promising therapeutic strategy for reducing GV in diabetes. In addition, multidisciplinary team approaches were reported to be remarkably associated with improvements in glycemic control [103, 104], which was highly recommend in the management of diabetes.

In recent years, neurodegeneration emerges as an early event in the pathogenesis of DR, and GV was reported to be a classical modifiable factor associated with all the stages of DR in T2DM [70]. Thus, retinal imaging for DR screening and better phenotyping of very early stages of DR might be instructive in taking solid measures to improve GV and prevent the progression of DR through targeting the neurovascular unit [105, 106].

### Pharmacological therapeutic strategies

Apart from the non-pharmacological therapeutic strategies, pharmacological therapeutic strategies are also crucial for improving GV and the cornerstone of diabetes management. A previous study investigated that basal insulin had more advantages than premixed insulin in reducing GV and hypoglycemia [107]. Meanwhile, insulin degludec and glargine contributed to better HbA1c and TIR with reduced hypoglycemia in toddlers and preschoolers with T1DM [108]. Furthermore, Nakamura et al. concluded that insulin degludec could lead to a smaller day-to-day variability of FPG compared with insulin glargine in T1DM patients [109]. Dipeptidyl peptidase-4 inhibitors (DPP4i) mainly increasing meal-stimulated insulin secretion are the important antidiabetic drugs. Early DPP4i intensification could reduce GV and ultimately contribute to the delay of insulin initiation [110]. Nagayama et al. explored the impact of vildagliptin, a DPP4i, on the parameters of blood glucose, and found that DPP4i could lead to a significant decrease in the median HbA1c and vascular complications in T2DM patients [111]. Additionally, a randomized controlled trial showed that anagliptin was remarkably superior in improving MAGE and TIR in T2DM patients [112]. Notably, a further study indicated that sitagliptin had an advantage in improving TIR in Japanese patients with early-stage T2DM and a lower BMI, whereas dapagliflozin was significantly superior in achieving TIR in the patients with a higher BMI [113]. In addition to directly influencing the variables of GV, dapagliflozin as an adjuvant therapy to basal insulin also exhibited a significant decrease in fluctuations of blood glucose in Japanese patients with T2DM [114]. Consistently, increasing evidence implicated that sodium-glucose cotransporter inhibitors (SGLT2i) as an add-on therapy to insulin showed a positive effect on improving glycemic control in patients with T1DM [115–117]; however, SGLT2i were correlated with higher odds of diabetic ketoacidosis [118], which weakened the benefits of SGLT2is on glycemic control. Further multicenter and randomized studies are warranted to explore more optimal therapeutic strategies for improving GV.

## Summary and further perspectives

With the advance of new glucose monitoring technologies, numerous variability indices of GV have been proposed, which not only plays an important role in DR, but also may be useful to predict it. However, there is still lack of a clear universal definition for assessing GV in clinical practice, and the exact mechanisms through which GV mediates DR are not fully understood. Thus, further research is warranted to better decipher and define GV, as well as explore the explicit mechanisms linking GV and DR.

Taken together, clarifying clear definitions and taking potential therapeutic strategies for improving GV contributes to helping the clinicians for the prevention and better clinical management of DR.

## Data Availability

Not applicable.

## Abbreviations

DR: Diabetic retinopathy
GV: glycemic variability
NPDR: non-proliferative diabetic retinopathy
PDR: proliferative diabetic retinopathy
T2DM: type 2 diabetes mellitus
HbA1c: hemoglobin A1c
FPG: fasting plasma glucose
DCCT: Diabetes Control and Complications Trial
T1DM: type 1 diabetes mellitus
SMBG: self-monitoring of blood glucose
CGM: continuous glucose monitoring
rtCGM: real-time continuous glucose monitoring
iCGM: intermittently viewed continuous glucose monitoring
FGM: flash glucose monitoring
PPG: postprandial glucose
SD: standard deviation
CV: coefficient of variation
SV: successive variation
VIM: variation independent of the mean
ARV: average real variability
IQR: interquartile range
MAGE: mean amplitude of glycemic excursions
CONGA: Continuous Overall Net Glycemic Action Index
TIR: time in range
MODD: mean of daily differences
HBGI: high blood glucose index
LBGI: low blood glucose index
ADRR: average daily risk rates
ACCORD: Action to Control Cardiovascular Risk in Diabetes
VADT: Veteran Affairs Diabetes Trial
TODAY: Type 2 Diabetes in Adolescents and Youth
LADA: latent autoimmune diabetes of the adult
EWDR: early worsening of diabetic retinopathy
RMCs: retinal Müller cells
HIF-1α: hypoxia-inducible factor-1α
DPP4i: dipeptidyl peptidase-4 inhibitors
SGLT2i: sodium-glucose cotransporter inhibitors
